# Promoting the health of migrant seasonal agricultural workers in rural Minnesota using a whole-person health approach: a pilot project

**DOI:** 10.3389/fpubh.2025.1518686

**Published:** 2025-04-02

**Authors:** Shahidali Jaffer, Jessica Hane, Margaret Eckerstorfer, Robin Austin, Jonathan D. Kirsch

**Affiliations:** ^1^Department of Medicine, University of Minnesota Medical School, Minneapolis, MN, United States; ^2^Office of Clinical and Academic Affairs, University of Minnesota, Minneapolis, MN, United States; ^3^Center of Spirituality and Healing, University of Minnesota Nursing School, Minneapolis, MN, United States

**Keywords:** migrant workers, health promotion, whole person health, mHealth app, community based organization

## Abstract

**Introduction:**

Promoting the health of the migrant and seasonal agricultural worker (MSAW) community is a unique challenge due to the particular social and economic barriers this community faces. Whole-person health assessments can aid in better understanding the specific needs of a community by accounting for social determinants of health (SDOH) and recognizing and leveraging a community’s strengths to assist in improving community health. To better optimize services provided at outreach health fairs for the MSAW community, the University of Minnesota performed comprehensive whole-health assessments using the mobile health (mHealth) application MyStrengths+MyHealth (MSMH). Results from these assessments were used to augment provided resources at future health events and create new community-specific interventions.

**Methods:**

In August 2022, participants receiving healthcare services from the Mobile Health Initiative (MHI) were asked to complete the MSMH survey. This whole-person health assessment comprises 42 health concepts that utilize participants’ self-reported strengths, challenges, and needs. Participants were provided a financial incentive to complete the assessment.

**Results:**

Thirty-one participants completed the MSMH survey. The majority were between the ages of 45–64 (35.5%) and self-identified as female (80.6%), white (64.5%), Hispanic/Latinx (93.5%), married (48.4%), and high school educated (41.9%). Overall, participants had many more strengths than challenges and needs; however, challenges were noted in the *Vision* (35.5%) and *Income* (29.0%) domains, leading to targeted interventions to improve these areas at future health outreach events.

**Conclusion:**

Utilizing a whole-person health assessment framework such as MSMH can result in a more nuanced understanding of a community, including its unique strengths, needs, and challenges. This information can be invaluable for health outreach groups seeking to promote community health by identifying upstream factors contributing to health outcomes. For the MSAW community in Minnesota, MSMH survey data were used to promote community health by increasing services, connecting individuals with community resources, and establishing vision and oral health programs.

## Introduction

Migrant and seasonal agricultural workers (MSAWs) are individuals whose primary employment is in agriculture and who establish temporary residence in locations across the United States following the harvest seasons (1). Estimates of the number of MSAWs in the United States range from 1–3 million individuals (2). MSAWs are typically Hispanic/Latino, with most of these individuals hailing from Mexico. Challenges for MSAWs and their families are numerous, primarily centering on the fact that agricultural workers represent one of the most economically disadvantaged communities in the United States (3). There are also social challenges, such as substandard and overcrowded housing options for MSAWs and their families (4). Ultimately, the social and economic realities of life for MSAWs and their families lead to numerous downstream adverse health outcomes. Mental health disorders, including post-traumatic stress disorder, depression, and anxiety, are higher in MSAWs than in the general population (5). Rates of diabetes, hypertension, and obesity are similarly elevated in this group compared with the general population (6). Taken as a whole, the upstream socioeconomic factors leading to negative health outcomes for MSAWs and their families mean that providing healthcare for this population is a complex challenge that must consider the unique challenges resulting from a migratory lifestyle (7).

Given the complicated interplay between social determinants of health (SDOH) and health outcomes, various models have been used in the public health literature to explore the numerous factors leading to health and disease. In 2017, the CDC introduced Public Health 3.0, a new paradigm for improving public health that called upon collaborations between the traditional healthcare system and community partners to improve social determinants of health (8). Further building upon Public Health 3.0 is “whole-person health,” which broadly considers a person’s environment, physical health, psychosocial aspects, health behaviors, and, importantly, strengths – when evaluating one’s health (8). In this model, strengths are defined as assets of individuals or families that are needed to maintain or improve their well-being in the face of short-and long-term stressors. Emerging data shows that individuals with higher levels of self-reported strengths can use their resilience to offset health challenges and improve health outcomes (9). Therefore, an assessment of whole-person health can provide context to an individual’s overall health and enable health professionals to address specific needs for individuals and communities beyond physical health. Whole-person health encourages a broader, holistic approach that recognizes the interconnectedness of health with various facets of society – including the traditional healthcare system – and ultimately aims to improve population health outcomes and well-being (8, 9). Ultimately, a whole-person health model can enable care teams to use a community-centric approach to inform decisions to address a population’s specific needs, emphasizing SDOH and considering an individual’s and community’s strengths (8–9).

Considering the complicated relationships between social factors and downstream health consequences for MSAWs and their families, approaching health promotion in this community through a “whole person health” lens makes intuitive sense; however, obtaining consistent data from this community can be challenging due to logistical barriers. Informatics tools, such as mobile health apps (mHealth apps), can provide a means to collect data anywhere at any time and represent an innovative approach to working with the MSAW community. A growing body of evidence has shown the effectiveness of mHealth apps in allowing patients to better self-manage medical conditions like diabetes and obesity, and extends to mental health conditions like depression and anxiety (10, 11). Data from mHealth apps and consumer-generated health data (CGHD) can also provide valuable data for healthcare organizations between clinical care visits (12). These data may be shared across settings and platforms with providers. Informatics also offers tools that may advance whole-person assessments and strengths-based healthcare. Over the past 20 years, several studies have implemented mHealth apps to promote the health of MSAW communities. However, the efficacy and long-term durability of these interventions have been limited due to difficulty in patient follow-up (13). One example of an informatics tool designed to be administered in a mobile setting and incorporating the values of whole-person health is MyStrengths+MyHealth (MSMH). The MSMH app is a mobile-optimized web-based mHealth app for consumers to assess comprehensive, holistic health across four domains: Environmental, Psychosocial, Physiological, and Health-related behaviors (14, 15).

In this study, we report the results from a project that used the mHealth application MSMH to improve the quality of services delivered at mobile health clinics for migrant seasonal agricultural workers (MSAWs) in Southern Minnesota. Survey results (*n* = 31) assessing the whole-person health of community members were used to tailor services for this community at subsequent health events, including an increased emphasis on connection with local resources/support and developing a new service line focused on vision health.

## Community context

In Minnesota, an estimated 15,000–20,000 seasonal agricultural workers arrive for work during the peak harvesting season from May to October each year (16, 17). Most of these workers are employed by food processing plants in Southern Minnesota rather than seasonal agricultural work. These workers usually split their time between Texas and Minnesota and originate from Central America (17). In June 2020, the University of Minnesota launched the Mobile Health Initiative (MHI) to advance health equity by bringing together health professionals with community organizations to reach underserved communities (18). Since its inception, MHI has served over 3,000 individuals throughout Minnesota in collaboration with local community-based organizations (CBOs) to deliver a range of urgently needed healthcare services across the state. MHI sponsors health events held at trusted community sites and provides services that CBOs request, including 913 individuals at 18 events in Southern Minnesota specifically focused on improving the health of MSAWs and their families. These events began in the summer harvest season of 2018. Services provided at MHI events included screening for hypertension, diabetes, and hypercholesterolemia, immunizations, dental and vision evaluations, medication refills, and physical examination services.

While these services were well-received, informal feedback from community members suggested that interventions focused solely on healthcare delivery were insufficient for this community’s needs. Thus, to better understand the MSAW community in Southern Minnesota, MHI implemented survey assessments using the MSMH tool at three community health events during the summer of 2022.

### MSMH survey instrument

**Figure 1:**
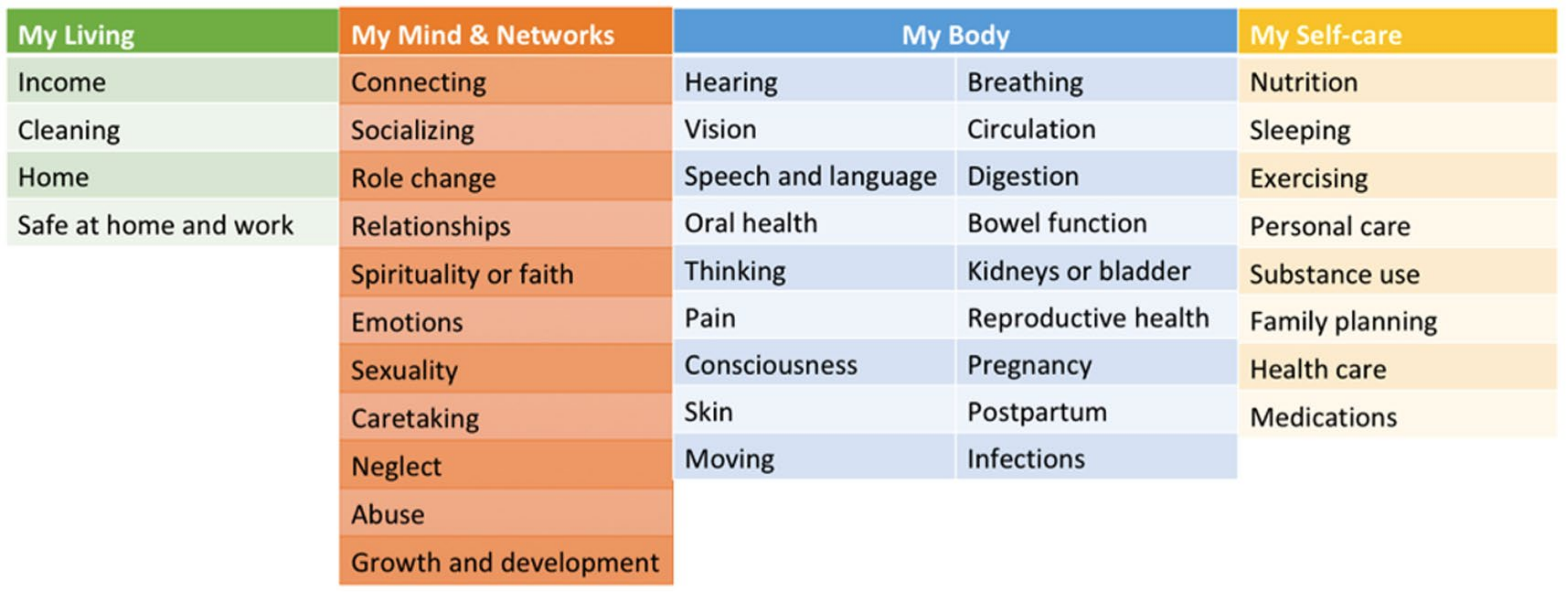
The list of domains and concepts used in MyStrengths+MyHealth. MSMH categorizes health into domains and concepts. Participants are asked about signs/symptoms related to each of these concepts. The Problem Rating Scale for Outcomes determines whether the concept is a “strength,” the Problem Classification Scheme is used to determine “challenges,” and the Intervention scheme is used determine if individuals have “needs” that would benefit from intervention.

MSMH was developed to enable self-reporting of individual strengths, challenges, and needs. The MSMH assessment uses the Omaha System, a multidisciplinary standardization of terminology, with consumer-facing terms that have been expert and community-validated and written at the 5th-grade reading level: Simplified Omaha System Terms (19). The Omaha System and its Simplified Omaha System Terms translation consist of three validated and reliable instruments: The Problem Rating Scale for Outcomes, the Problem Classification Scheme, and the Intervention Scheme (20). The Omaha System describes and quantifies all of an individual’s health in 42 discrete, taxonomic problem concepts arranged within four domains: environmental (4 concepts), psychosocial (12 concepts), physiological (18 concepts), and health-related behaviors (8 concepts) (20). Each concept is first defined by a unique set of signs/symptoms, ranging from 3–17 signs/symptoms per concept. In the MSMH application, users select the Problem Rating Scale for Outcomes Status scale responses, and a “strength” is calculated as a score of 4–5 out of 5. Figure 1 includes all the domains and concepts included in the MSMH assessment.

The Problem Classification Scheme concept also incorporates signs and symptoms termed “Challenges.” Each concept has a range of unique challenges, ranging from 3 to 16, and users can select any, all, or “None apply.” Finally, the Intervention Scheme is called “Needs” in MSMH. It describes problem-specific actions, and users can identify if they may need assistance in any of the following categories: teaching, guidance, counseling (info/guidance), treatments and procedures (hands-on care), case management (care coordination), and surveillance (check-ins). Users can select any, all, or “No Needs” if none apply to that concept (14). In conclusion, using the MSMH tool, individuals can self-report health challenges, rate their health overall, and identify if they have any health needs – across 42 health concepts.

### Data collection and analysis

In August 2022, MHI used the MSMH tool to collect de-identified data from the MSAW community in southern Minnesota during two health events. Participants voluntarily completed the MSMH survey, and Spanish-speaking volunteers were available to help them with survey-related questions. The survey instrument took participants approximately 20–30 min to complete in its entirety. Upon completion of the survey, participants received $20 Visa gift cards. The Institutional Review Board of the University of Minnesota approved this project. MSMH data were analyzed using SPSS Version 18.0. The data examined all participants’ strengths, challenges, and needs.

## Results

**Figure 2:**
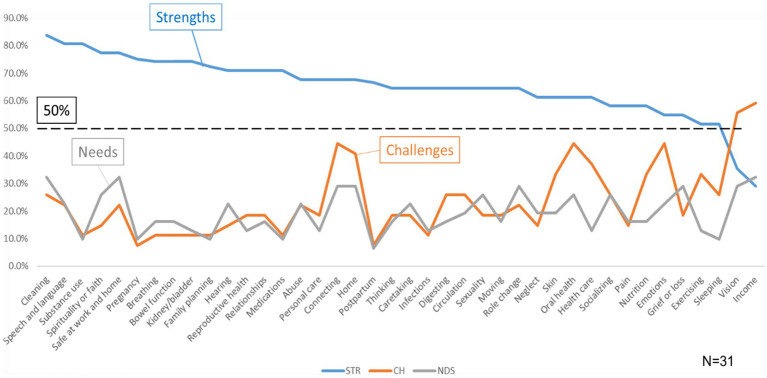
Overall results of strengths (STR), challenges (CH), and needs (NDS) by concept. In general, strengths were noted to have an inverse relationship with challenges and needs, with a few exceptions including personal care and connecting.

Overall, 31 participants completed the MSMH survey. The majority were 45–64 (35.5%), female (80.6%), white (64.5%), Hispanic/Latinx (93.5%), married (48.4%), and with high school education (41.9%). Participants had many more strengths than challenges and needs, with a majority of strengths above the 50% threshold, except *Vision* (35.5%) and *Income* (29.0%). Figure 2 shows how strengths, challenges, and needs varied amongst the surveyed concepts.

### Strengths

**Figure 3:**
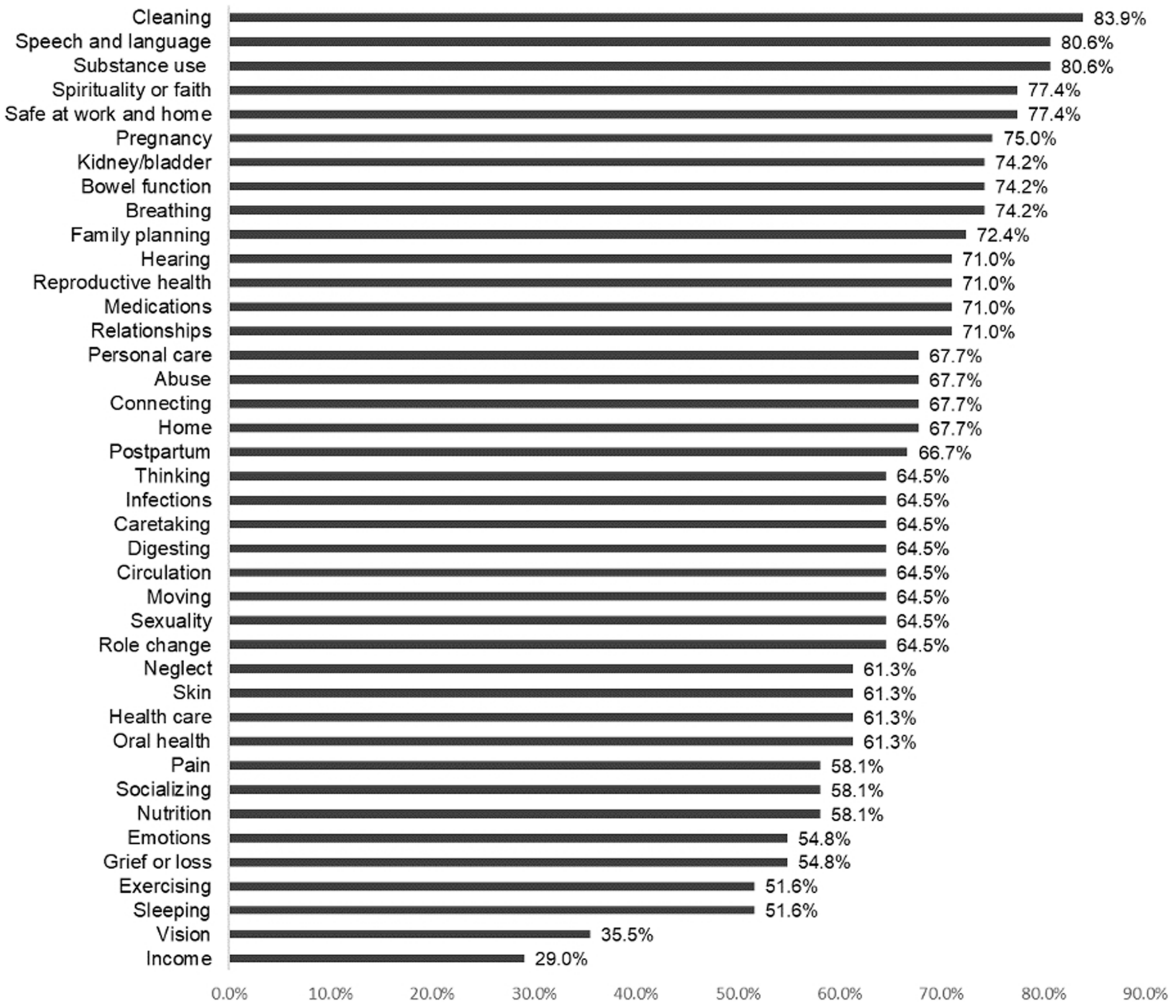
Overall strengths by concept. Participants had a high degree concordance with respect to reported strengths, with cleaning, speech and language, and substance use as most commonly identified strengths.

The most frequent strengths were from *Cleaning* (83.9%), followed by *Speech and language* (80.6) and s*ubstance use* (80.6%) (meaning no substance use). Figure 3 shows the identified community strengths across all concepts.

### Challenges

**Figure 4:**
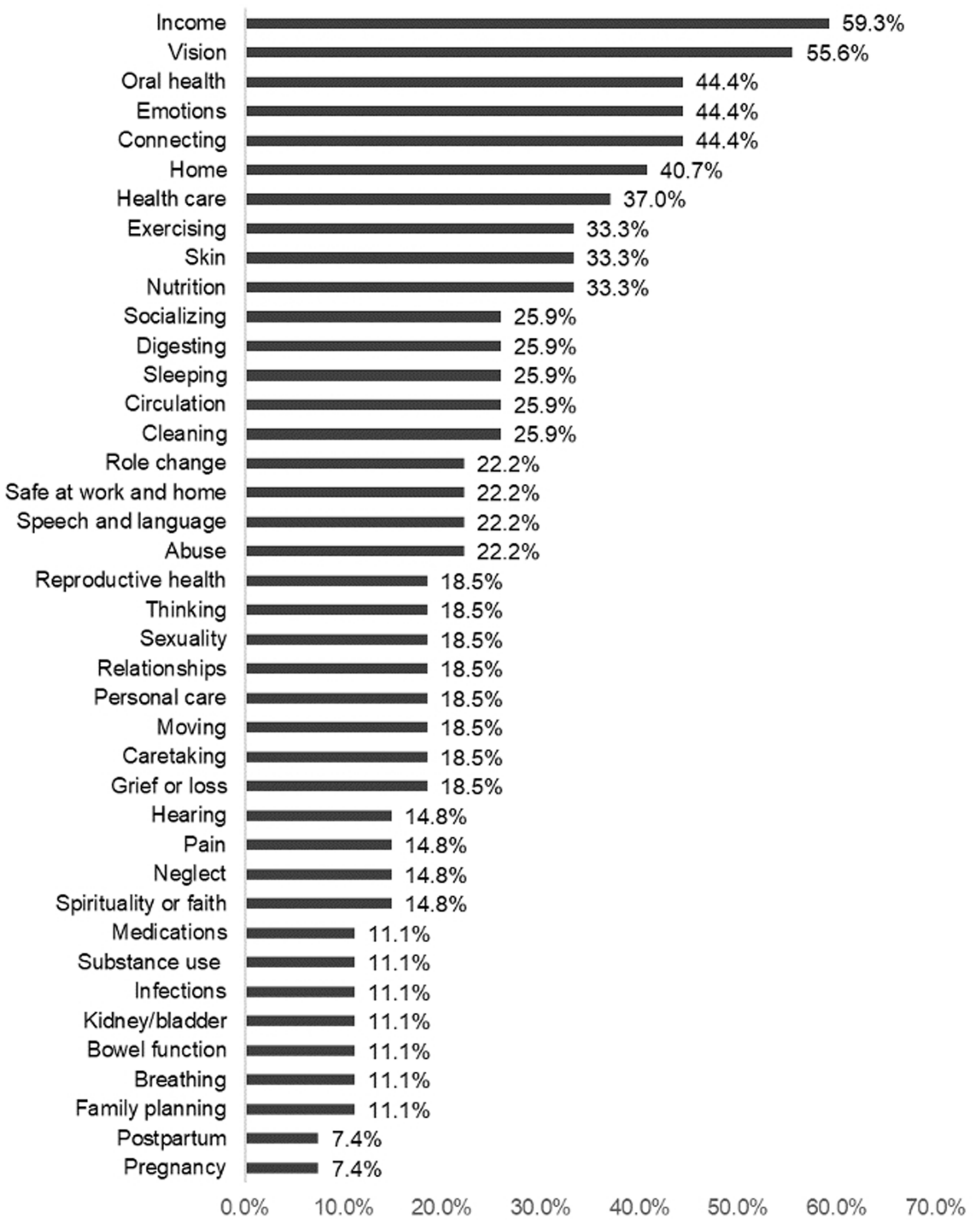
Overall challenges by concept. Compared to strengths, we found less concordance overall with reported challenges in the population surveyed, with income and vision being the most identified challenges.

The most frequently reported challenges came from the Domains of *Income* (59.3%), followed by *Vision* (55.6%) and *Connecting*, *Emotions*, and *Oral health* (44.4%). Figure 4 shows how challenges varied for survey respondents across concepts. The most frequently reported challenges within each domain included *hard-to-see small print* (33.3%) from the *Vision* concept, followed by *tired* (29.6%) from the *Emotions* concept and *not enough income (25.9%) Income* concept.

### Needs

**Figure 5:**
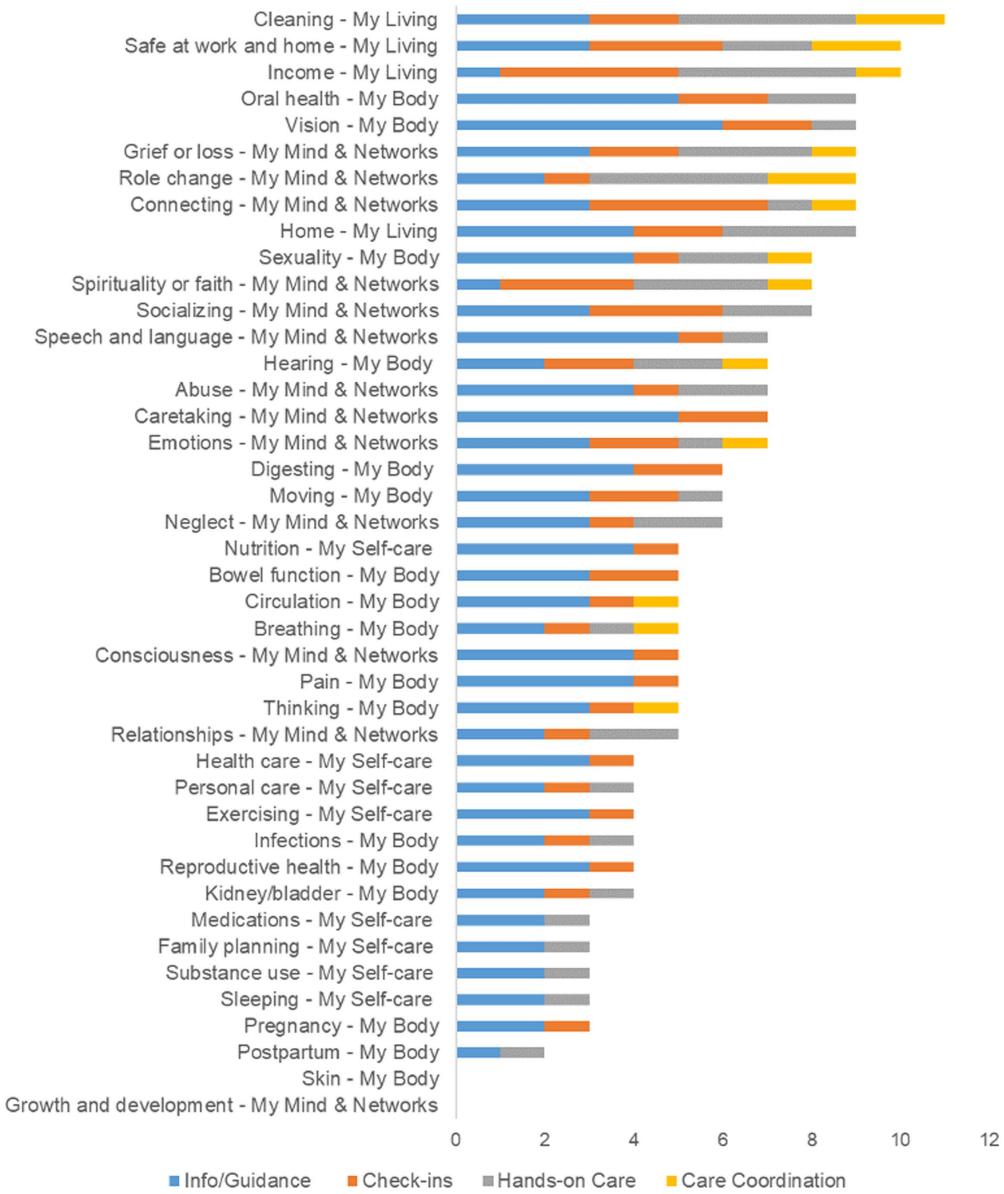
Overall needs by concept and domain. Using the intervention scheme, participants were asked what type of assistance would be most helpful for the needs identified in the MSMH survey. Information and guidance were the most commonly requested service regardless of domain and/or concept.

The most requested Needs were in the *Cleaning* concept (10), followed by *Safe at home and work*, and *Income*. Figure 5 shows how patient reported needs varied across concepts. The most frequent category of need across all concepts was info/guidance (48.5%), followed by check-ins (23.9%), Hands-on Care (21.0%), and coordination (6.6%).

## Discussion

### Strengths

Overall, participants in this pilot identified many more strengths than challenges and needs. The most frequent self-reported strengths were *Cleaning, Speech and Language*, and *Substance use* (i.e., no substance use). Some of these results specifically reflect the demographics of individuals who attended our health events and took this survey (i.e., mothers and older women), who are generally both at lower risk for substance use disorders and more engaged in spiritual life. In particular, we highlight the importance of the Spanish language as a strength in this community, reflecting the importance of Spanish language concordance for MSAW workers, their communities, and families.

In this community, Spirituality and Faith are other important strengths. It is estimated that 80% of Mexicans and 55% of Latinos in the United States identify themselves as Roman Catholics (3). In Latin American immigrants, religiosity has been shown to mediate stress and symptoms of depression (21). Additionally, there are also small studies showing an inverse relationship between risks of substance use disorder in patients who do have a strong faith background (22, 23). Faith and spirituality are also associated with increased resiliency, which may partly explain how MSAWs can cope with numerous challenges related to a migratory lifestyle (24). Finally, with shared religion often comes community, and many indigenous and non-indigenous people from Central America working as MSAWs are part of close-knit rural communities in their home countries that frequently follow them upon migration to the USA. These “hometown” networks are an essential aspect of culture and often allow migrant communities to share resources, advice, spiritual and cultural traditions, and other support (25). Thus, the strengths of the MSAW community, as identified by the MSMH instrument, reflect the importance of faith for MSAWs and are consistent with the protective effects of faith on substance use and resiliency seen in other data.

### Challenges

The most common challenges identified by the survey participants were difficulties with *Income, Vision,* and *Emotions*. The overall challenge of *Income* for this community is consistent with our understanding of the MSAW community, with a median pay of $29,680 per year as of 2021, making this one of the most socially and economically disadvantaged communities in the United States. Lack of income leads to numerous consequences, including food insecurity, limited transportation options, housing instability, and an inability to access healthcare or medications due to costs and transportation (2, 3, 26).

In addition to income, vision was another challenge identified for the MSAW community in this survey. The long-term impact of visual impairment and preventable causes of blindness is now a matter of national public health concern, with over 3.4 million Americans currently affected-furthermore, this number is expected to double by 2030 (3). Unfortunately, the burden of eye disease is concentrated on populations experiencing barriers to care, and in no group is this more pronounced than for migrant agricultural workers, who experience some of the highest rates of visual impairment in the United States (3, 26). In addition to common visual conditions like diabetic and hypertensive retinopathy seen across United States communities, MSAWs also experience unique challenges to vision, including exposure to agricultural chemicals, wind, dust, allergens, and UV light exposure (27). Due to economic challenges and limited healthcare access, MSAWs and their families are less likely to access and afford vision care services (28).

Finally, the assessment identified the concept of *Emotions* (Mental health) as another challenge. Indeed, behavioral health conditions are widespread in migrant agricultural communities (21). Baseline rates of depression and anxiety disorders are higher in this group than in the general population (5, 29, 30). This, combined with embedded cultural norms of faith, familyism, and self-reliance, results in under-recognition and under-treatment of mental health disorders and makes the morbidity of mood disorders disproportionately high in this population (31).

### Needs

The most common need was related to “Hands-on care” for cleaning. The self-reported needs are consistent with other studies in various contexts (e.g., connecting with others and managing anxiety and depression) (7, 32). This is also consistent with previous findings highlighting that concepts with higher reported challenges also have more self-reported Needs (e.g., *Income* and *Emotions*) (33, 34).

### Impact

The overall impact of these survey results on mobile clinic operations was to broaden the services provided at health fair events beyond health screenings to interventions that better target the specific social determinants of health impacting the health of migrant farmworkers and their families in collaboration with our community partner.

One of the primary needs identified by the community in this survey was income. Though we could not directly intervene in this need, we elected to focus our efforts on connecting the community with local programs and organizations capable of bridging the resource gap, as it is well-known that migrant workers have difficulty accessing state and federal programs due to administrative and logistic barriers (1). Via QR codes shared widely at our health events and by the invitation of state and community-sponsored organizations to table at health fair events, community members can now connect locally with food pantries and local farmers’ markets, social services (including health insurance, occupational health/safety, and housing assistance), transportation, and childcare resources.

Two other community-identified needs were vision services and oral health. In response, MHI invested in equipment, training, and recruitment of eyecare professionals to screen community members for refractive error, diabetic retinopathy, or advanced die disease requiring ophthalmology follow-up. Additionally, through collaborations with local organizations, glasses can be provided to patients at no cost. These vision screening events have become the most popular and requested services MHI provides at health events. Moving forward, our focus is on broadening MHI’s partnerships with local eye clinics and hospitals to coordinate follow-up care for those needing referrals, as follow-up care, medications, and surgery for eye-related problems remain a significant barrier to vision care. Regarding oral health, MHI has also partnered with the School of Dentistry to provide dental screenings, education, and limited interventions for adults and children seen at health events. Similar to our vision platform, mobile dental clinics have become extremely popular with community members (and in particular, for children).

An area of less success for MHI has been interventions targeting the high rates of behavioral health disorders in MSAWs and their families. Though evidence-based stress and anxiety management practices via mobile applications have been broadly shared at events, along with specific resource guides that are culturally competent, patients attending the mobile health clinic, in general, have been less apt to engage with volunteers regarding these issues. This may reflect the physical limitations of mobile health events, where less privacy is available, and attendees often know each other. This continues to be an area of focus for the MHI team moving forward. However, our attempts in this area have also signified that mobile health events can be challenging locations to have these more delicate conversations.

In conclusion, MSMH results provided the MHI team with actionable data that increased resources at health events focused on improving SDOH and empowered the expansion of medical services like vision and dental services for migrant workers and their families. Survey results also allowed our team to better triage services and resources provided as part of the mobile health clinic, adjusting focus to services (e.g., vision and dental) that were most important – as determined by the community itself.

### Limitations

This study had some notable limitations. Most MSAWs are younger men, while this study was composed of primarily middle-aged women. This likely reflects selection bias due to hosting our events in childcare centers near migrant camps with a focus on pediatric health, and being unable to hold events late enough in the evening for those field workers to attend routinely. This small sample size is, therefore, also only generalizable to this population.

### Future directions

Use of patient-facing tools, such as MSMH, can effectively screen communities for social determinants of health and comprehensively assess a community’s strengths, challenges, and needs. This approach benefits from using validated and standardized terminology, allowing for robust data analysis and the potential to imbed/incorporate results directly into the electronic health record. For groups or organizations working with various communities, tools like MSMH allow organizations to tailor their health outreach efforts better, targeting community-specific interventions promoting health equity. Based on positive feedback from community-based organizations and community members alike, MHI plans to expand use of MSMH in the future to assess the needs of additional underserved populations across the state of Minnesota.

Finally, while our project focused on using MSMH as a community-level tool, there is also a role for using it at an individual level. For example, following an individual’s strengths, challenges, and needs over time could help a medical provider individualize their approach to the patient’s care—particularly when attempting to understand the upstream factors leading to health consequences.

## Data Availability

The raw data supporting the conclusions of this article will be made available by the authors, without undue reservation.
