# Correction: Promoting the health of migrant seasonal agricultural workers in rural Minnesota using a whole-person health approach: a pilot project

**DOI:** 10.3389/fpubh.2025.1675896

**Published:** 2025-09-30

**Authors:** Shahidali Jaffer, Jessica Hane, Margaret Eckerstorfer, Robin Austin, Jonathan Kirsch

**Affiliations:** ^1^Department of Medicine, University of Minnesota Medical School, Minneapolis, MN, United States; ^2^Office of Clinical and Academic Affairs, University of Minnesota, Minneapolis, MN, United States; ^3^Center of Spirituality and Healing, University of Minnesota Nursing School, Minneapolis, MN, United States

**Keywords:** migrant workers, health promotion, whole person health, mHealth app, community based organization

Authors Jessica Hane and Margaret Eckerstorfer were omitted as authors in the published article. The correct author list reads:

Shahidali Jaffer^1^^*^, Jessica Hane^1^, Margaret Eckerstorfer^2^, Robin Austin^3^, and Jonathan Kirsch^1^

^1^Department of Medicine, University of Minnesota Medical School, Minneapolis, MN, United States

^2^Office of Clinical and Academic Affairs, University of Minnesota, Minneapolis, MN, United States

^3^Center of Spirituality and Healing, University of Minnesota Nursing School, Minneapolis, MN, United States

The Author Contributions Statement has been corrected to read:

SJ: Writing – original draft, Writing – review & editing. JH – Conceptualization, Writing – review & editing. ME: Conceptualization, Writing – review & editing. RA: Writing – original draft, Writing – review & editing. JK: Writing – original draft, Writing – review & editing.

The original version of this article has been updated.

